# Reconstruction of Soft-Tissue Defects at the Foot and Ankle after Oncological Resection

**DOI:** 10.3389/fsurg.2016.00015

**Published:** 2016-03-08

**Authors:** Andrej Ring, Pascal Kirchhoff, Ole Goertz, Bjorn Behr, Adrien Daigeler, Marcus Lehnhardt, Kamran Harati

**Affiliations:** ^1^Department for Plastic and Hand Surgery, Burn Center/Sarcoma Reference Center, Ruhr-University Bochum, Bochum, Germany

**Keywords:** sarcoma, reconstructive surgical procedures, lower extremity, microsurgery, flap

## Abstract

**Introduction:**

Solid malignancies at the foot and ankle region are rare and include mainly soft-tissue sarcomas, bone sarcomas, and skin malignancies. Complete surgical resection with clear margins still remains the mainstay of therapy in these malignancies. However, attainment of negative surgical margins in patients with locally advanced tumors of the foot and ankle region may require extensive surgery and could result in loss of extremity function. In these circumstances, plastic surgical techniques can frequently reduce functional impairment and cover soft-tissue defects, particularly in cases of large tumor size or localization adjacent to critical anatomic structures, thereby improving the quality of life for these patients. The aim of this article is to illustrate the various treatment options of plastic surgery in the multimodal therapy of patients with malignant tumors of the foot and ankle region.

**Materials and methods:**

This article is based on the review of the current literature and the evaluation of the author’s own patient database.

**Results:**

The local treatment of malignant extremity tumors has undergone major changes over the last few decades. Primary amputations have been increasingly replaced by limb-sparing techniques, preserving extremity function as much as possible. Although defect coverage at the foot and ankle region is demanding due to complex anatomical features and functional requirements, several plastic surgical treatment options can be implemented in the curative treatment of patients with malignant solid tumors in this area. Soft-tissue defects after tumor resection can be covered by a variety of local flaps. If local flaps are not applicable, free flap transfers, such as the anterolateral thigh flap, parascapular flap, or latissimus dorsi flap, can be utilized to cover nearly all kinds of defects in the foot and ankle region.

**Conclusion:**

Soft-tissue reconstruction in the foot and ankle region is a vital component of limb-sparing surgery. It enables complete resection of locally advanced tumors and subsequent adjuvant radiotherapy. Modern plastic surgical techniques should, therefore, be integrated in the multimodal treatment concept of malignancies in the foot and ankle region.

## Tumors of the Foot and Ankle Region

Malignant neoplasms on the foot and ankle make up about 4% of all bone and soft-tissue tumors of the body (1–3). Soft-tissue sarcomas, as rare tumors of mesenchymal origin, account for about 1% of all adult malignancies but are located in the extremities in about 60% of all cases (4). Regarding the content of soft tissues, the lower extremities are affected more frequently than the upper extremities, with a ratio of 3:1. Other rare malignancies with localization at the foot are, for example, tumors of the skin, such as melanoma, and giant cell tumors of the tendon-sheath, vascular sarcomas, and carcinoma metastases. The local treatment of soft-tissue sarcomas has undergone major changes over the last few decades. Primary amputations have been increasingly replaced by limb-sparing techniques, preserving extremity function as much as possible (3, 5). Interestingly, a recently published meta-analysis revealed that the width of negative surgical margins has no significant impact on overall survival (6). The data within the latter suggested that patients with clear margins had a better prognosis, but surgery to preserve functionality may result in very close margins without impairing survival. However, in patients with locally advanced soft-tissue sarcomas of the extremities, attainment of negative surgical margins may require extensive surgery and could result in a loss of extremity function. In these circumstances, plastic surgical techniques can frequently reduce functional impairment and cover soft-tissue defects, particularly in cases of large tumor size or localization adjacent to critical anatomic structures, thereby improving the quality of life for these patients. Whether limb preservation or ablative procedures are applied is also determined by the location of the tumor and the expectations and the functional demands of the patient.

## Oncological Treatment Strategies and Concepts

Contemporary treatment strategies should include concepts of complete resection with negative surgical margins in combination with plastic reconstructive surgery, especially considering the postoperative functional aspects. The factors that determine the resectability of sarcomas are mainly the tumor size and localization. Regarding surgical resectability, the localization of the tumor is of great relevance. Deep or subfascial localization is associated with a higher risk of local recurrence or distant metastasis when compared with superficial or epifascial localization (7–9). Regarding tumor size, large soft-tissue sarcomas are shown to have a diminished survival when compared with small tumors. However, large tumor size is found more frequently in high-grade tumors. Most survival studies have revealed that the tumor grade is the most significant prognostic factor (9, 10). Dedifferentiated and high tumor grade is also associated with higher invasiveness, resulting in extensive infiltration of the surrounding tissues.

In cases with extensive infiltration of functional structures, amputation still has to be considered as a treatment option in order to obtain clear margins (7, 11, 12). However, the fact that amputation as a curative treatment strategy for sarcomas in the extremities has no survival benefit when compared to limb-preserving surgical procedures should be taken into account. It has been shown that amputation, especially in combination with radiotherapy, has no advantage compared to resection (6). The use of reconstructive surgery and adjuvant radiation since the 1980s has been able to reduce the amputation rate to less than 20% (13–16). The incidence of local recurrences under limb preservation therapy has decreased to 10–15% in recent years; however, no change in terms of overall survival has been shown (17, 18). Local control rates for high-grade tumors amounted to about 90% and to 90–100% for low-grade sarcomas. Notably, high-grade tumors are associated with a higher risk of local recurrence (19, 20). Subsequently, local recurrence has been found to be associated with a diminished survival when compared with the primary disease (7, 21, 22). Nowadays, primary indications for amputations are fortunately rare. Primary amputations are indicated when negative margins are not otherwise attainable. However, whenever an amputation is deemed necessary to obtain local control of an extremity soft-tissue sarcoma, isolated limb perfusion should be considered. Tumor necrosis factor alpha-based isolated limb perfusion has been demonstrated to result in a limb salvage rate of 81% in patients with locally advanced extremity soft-tissue sarcoma who would have otherwise undergone amputation (23, 24). The second indication for primary amputation is the palliation of extensive ulcerative, bleeding, or odor-intensive tumors with complete loss of extremity function. A primary amputation must also be drawn early into consideration when satisfactory functional results cannot be expected from reconstructive techniques after extensive or disabling limb-sparing resections. In such cases, timely recovery and mobilization might be achieved earlier after primary amputation.

## Principles of Tumor Resection at the Foot and Ankle

The implementation of a radical surgical procedure in the distal region of the limb is often difficult due to a limited soft-tissue situation. Attainment of clear margins becomes much more difficult in distal tumors and can result in soft-tissue defects. Plastic surgical techniques remain particularly indispensable in the treatment of such distal tumors. The extent of resection after histological confirmation depends on the size, grading, and local relationship to functional structures. Decisions regarding further leading reconstructive procedures also depend on it. Negative margins should be the goal of surgical therapy. Performance of the appropriate margins in soft-tissue sarcomas is often complicated by the extended growth of the tumor. Margins to adjacent functional structures should be taken into account in the oncosurgical considerations. These functional structures are fascia, synovia, periosteum, and perivascular and perineural tissue. When there is tumor infiltration of such structures, en-block resection should be performed. The course of vascular structures should be taken into account during the resection to allow planning of reconstructive procedures by using pedicled or free tissue transplantation.

## Anatomical and Surgical Features

Whereas limb salvage has become the standard of care in the treatment of tumor in an extremity, the unique anatomy of the foot presents challenges in reconstructing a viable and functional limb. The defect coverage in the area of the foot and ankle region is demanding due to complex anatomical features and functional requirements. The skeletal stability of the foot and ankle region, the resilience of the soft tissues, and the preservation of sensation are at the forefront of reconstructive considerations. Here, profound knowledge of the anatomy of the foot and ankle area is essential for the development of a rational treatment algorithm for covering soft-tissue defects. Hidalgo and Shaw studied the arterial anatomy and the cutaneous nerve supply of the plantar skin extensively in the 1980s, providing guidelines of safe plantar incisions and flap design (25, 26). Prior to their work, flap designs on the plantar aspect of the foot were based on the concept that blood supply proceeds from the deep to the superficial tissue. Therefore, plantar flaps were usually raised subfascially requiring extensive dissection of the plantar soft tissue and, thus, resulting in severe foot and donor site morbidities (27). However, Hidalgo and Shaw demonstrated that local flaps at the plantar aspect could be designed with preserved sensitivity and abundant blood supply as reliable and safe alternatives without the need of subfascial dissection. Based on their findings, they further divided the foot into four major areas based on different requirements for reconstruction and the types of flaps available (28, 29). These are the proximal and distal weight-bearing plantar areas, the dorsum and the ankle region including the malleoli, the Achilles tendon, and the non-weight-bearing heel area. Furthermore, they provided a classification of foot defects and an algorithm of their coverage whose principles are still relevant. They preferred to cover soft-tissue losses less than 3 cm^2^ with local flaps in weight-bearing areas and with skin grafts in non-weight-bearing areas (Type I). Local flaps are more durable than skin grafts and allow normal weight-bearing on the reconstructed surface. Plain local flaps, such as V-Y flaps and transposition flaps, should be preferred to avoid a soft-tissue surplus, which could enhance strains on the scar at walking and, thus, increasing the risk of ulceration. Skin grafting represents a simple and safe procedure to cover small defects with a well-vascularized wound bed. However, skin grafts are usually suitable for smaller defects at the dorsum and non-weight-bearing areas at the instep but are fragile and can generate painful scars and ulcerations when transplanted on tenuous soft tissue. Therefore, Hidalgo and Shaw do not recommend skin grafting alone for larger defects. Such Type II defects, which are defined to be larger than 3 cm^2^ without bone involvement were preferred to be covered by free fasciocutaneous, free musculocutaneous flaps, or local flaps to ensure a durable soft-tissue coverage preventing recurrent ulcerations, improving the scar qualities, and reducing strains on the scars. Large tissue losses with bone involvement (Type III) were recommended to be reconstructed with free flaps or free osteocutaneous transfers if necessary. Here, adequate soft-tissue coverage also provides an essential base for following necessary surgical bone procedures. Bulky soft-tissue surpluses can be thinned out easily in future procedures after healing.

The reconstruction of the highly specific, thick, load-capable, and stress-resistant skin of the weight-bearing proximal and distal plantar area is more difficult, because the patient’s own tissue from other donor regions of the body does not often meet the requirements with regard to tissue quality and functional needs. By contrast, soft-tissue coverage of the dorsum of the foot and the ankle region is of thin and pliable skin with a delicate subcutaneous adipose tissue layer. This feature is often severe in surgical interventions, for example, in osteosynthetic care of ankle fractures. In this case, due to the peculiarity of the soft-tissue cover, wound complications with exposure of structures, such as tendons, joint capsule, bone, and hardware, can occur. Furthermore, the mobility of the soft tissue covering the foot and ankle is due to quite limited multiple zones of adherence. Consequently, defects in serving areas can rarely be treated by local tissue displacement. In such cases, tissue from other parts of the body must be free transplanted. However, the tissue units used for microvascularized transfer are often bulky and have to be thinned out and adapted in the course of correction operations for subsequent shoe care and esthetic perception. Furthermore, the fact that the free transplanted tissue is not innervated must be considered. This fact may stimulate the formation of pressure ulcers. The reconstruction of complex defects of the foot has experienced a steady improvement in recent decades. Achievements in the therapy have been kept particularly for innovative surgical techniques and a better understanding of the specific tissue situation and functional requirements of the foot and ankle area.

## Plastic Surgical Reconstruction Options

Tumor removal is often accompanied by major tissue defects. Furthermore, there might be an exposure of functional structures, such as bones, tendons, nerves, and blood vessels. Therefore, plastic surgical reconstruction methods must be an integral part of oncosurgical treatment concepts. Moreover, transfer of healthy tissue with adequate blood supply enables adjuvant radiation and chemotherapy, and might improve the effectiveness by improving oxygen nourishment in the tumor bed. A simple reconstructive procedure, such as skin grafting, is, therefore, not usual, since the resulting soft-tissue coverage is often insufficient for adjuvant radiotherapy. However, the reconstructive ladder should still be addressed when considering soft-tissue coverage at the foot and ankle area. Some local flaps have proven to be very efficacious in the treatment of foot and ankle defects. Small defects at the non-weight-bearing sole can be covered by simple cutaneous or myocutaneous V-Y flaps. Due to its reliable vascular supply, its proportions and the moderate donor site defect, the distally based sural flap has proven itself in the coverage of smaller defects at the hind foot and malleolar region (30–33). The dominant pedicle of the distally based sural artery flap is a branch arising from the popliteal artery descending from the popliteal fossa between the heads of the gastrocnemius muscle. It is accompanied by the medial sural cutaneous nerve from the tibial nerve and small venae comitantes. The sural artery flap is a fasciocutaneous flap located at the proximal dorsal area of the lower leg. Elevation of the flap proceeds proximally after visualization of the entrance of the dominant pedicle into the deep fascia in a subfascial plane until an adequate arc of rotation is achieved. Notably, the pivot point of the pedicle should be at least 5 cm proximal of the lateral malleolus in order to preserve the anastomoses with the peroneal artery. The maximum size of the distally based sural flap is limited because of its slight perfusion provided by the arterial network surrounding the sensory medial sural cutaneous nerve. Delayed distally based sural flaps can be raised larger and present an improved reliability but require a two-step surgical approach (34). Such delayed reverse sural flaps are particularly suited for small hind foot defects (35, 36). However, defects at the weight-bearing hind foot should ideally be reconstructed with sensible local flaps to avoid ulcerations. Mendieta et al. reported a case of a heel defect in which they connected the severed nerve end of a distally based sural flap with the intermediate dorsal cutaneous branch of the superficial peroneal nerve to give sensibility to the flap (37). Tan et al. recently reported a case series of such neurotized distally based sural flaps in 14 patients where all flaps survived and two-point discrimination achieved at least 14 mm after 6 months (38). The neurotized sural flap represents an interesting modification for the sensory reconstruction of weight-bearing hind foot defects and upcoming studies deserve attention. In the armamentarium of local flaps, the instep-island flap based on the medial plantar neurovascular pedicle provides another option for the sensory soft-tissue reconstruction of small hind foot defects (39, 40). The instep-island flap occupies the skin of the non-weight-bearing instep between the first metatarsal head and the distal portion of the heel. The flap is elevated distally deep to the plantar fasica and dissection continues proximally toward the medial plantar artery and nerve. Here, the medial plantar nerve can be split from distal to proximal in order to preserve the sensitivity of the flap and the medial foot (41, 42). The instep-island flap is suited for smaller defects, and the donor site must usually be transplanted with a skin graft.

**Figure 1:**
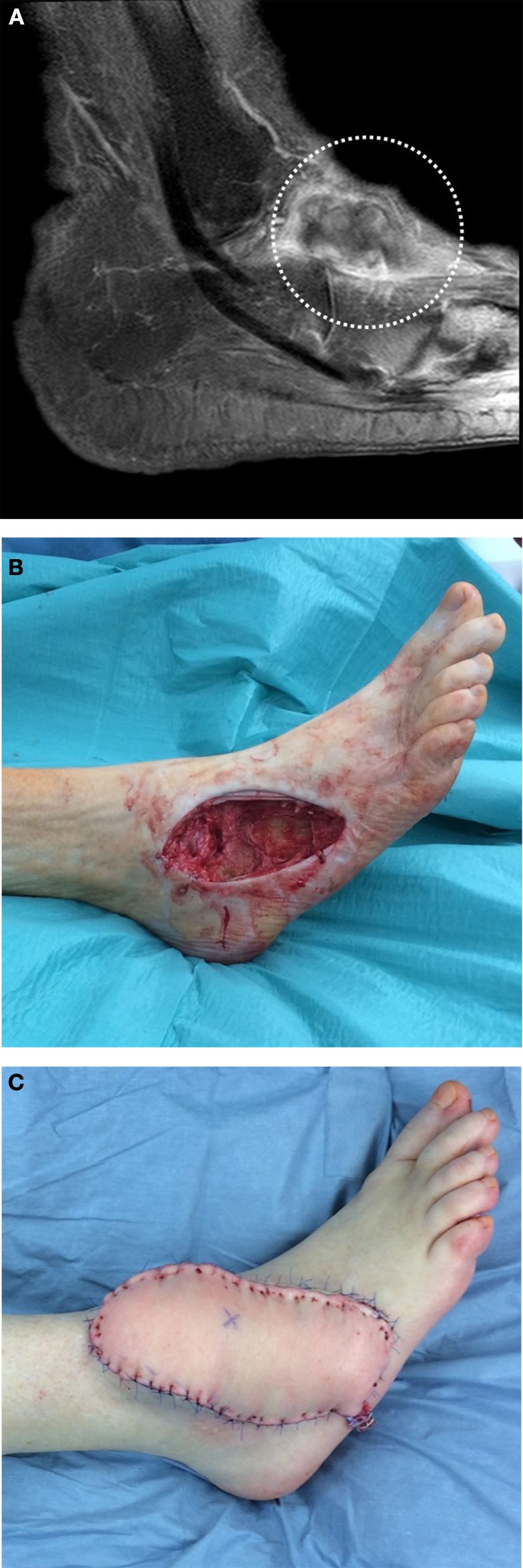
**A 77-year-old female was presented after incomplete resection of a liposarcoma G2 on the lateral ankle of her right foot**. The initial tumor localization is shown by MRI image **(A)**. A two-stage reconstruction of the defect after extensive resection **(B)** was done by free transplantation of an adipocutaneous flap **(C)** from the anterolateral region of the thigh (ALT flap).

**Figure 2:**
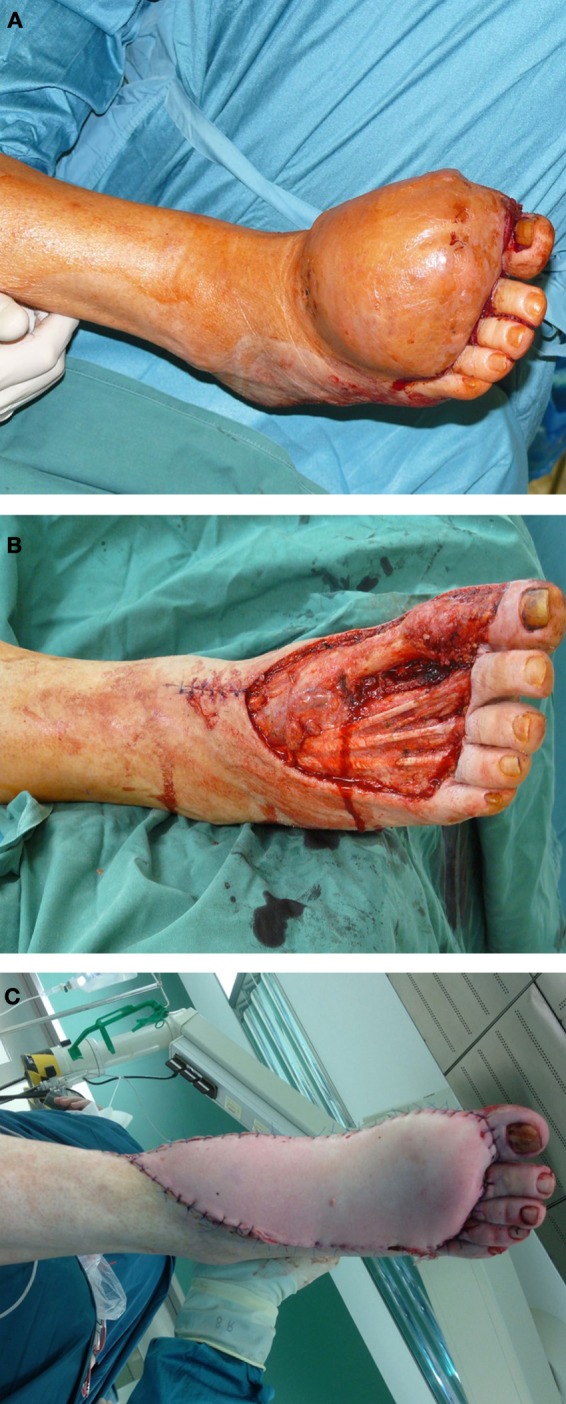
**A 62-year-old male presented with first diagnosis of a myxoid fibrosacoma G2 at the dorsum of his right foot (A)**. After oncological tumor resection, the defect was reconstructed with a free fasciocutaneous ALT flap from the thigh **(B,C)**.

**Figure 3:**
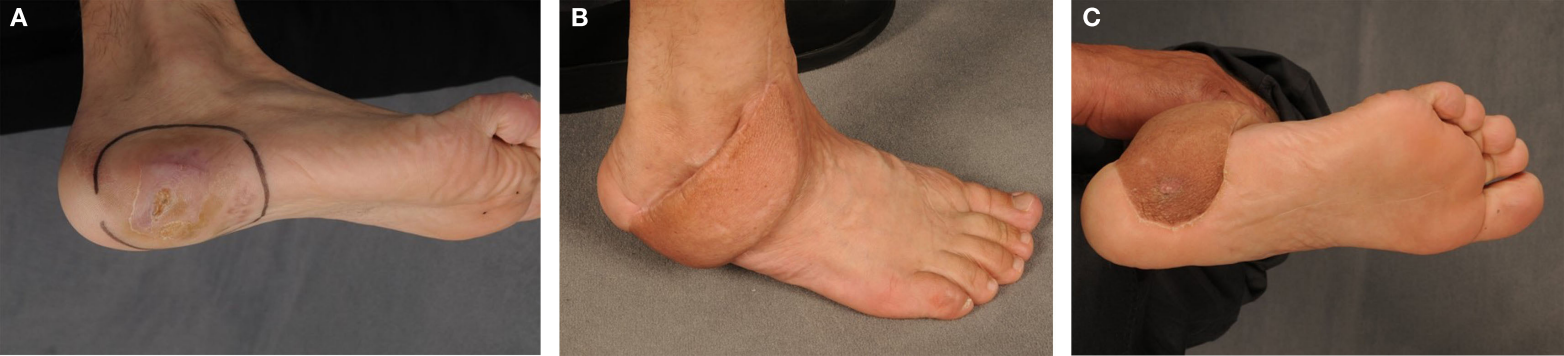
**A 49-year-old male with a clear cell sarcoma G2 localized on his right foot (A)**. The patient was transferred to our department for oncological resection and plastic reconstruction after an incomplete tumor resection. The coverage of the soft-tissue defect about the load-exposed part of the sole was performed by a free fasciocutaneous flap **(B,C)** from the parascapular area.

**Figure 4:**
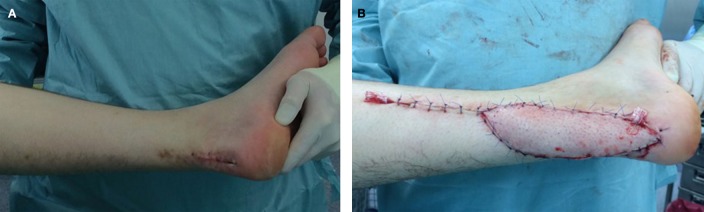
**A 54-year-old male with infiltrative myxoid fibrosarcoma G2 of the Achilles’ tendon of his left leg and R2 status after foreign surgery (A)**. Reconstructive procedure after oncological resection was performed by use of a free parascapular flap **(B)**.

**Figure 5:**
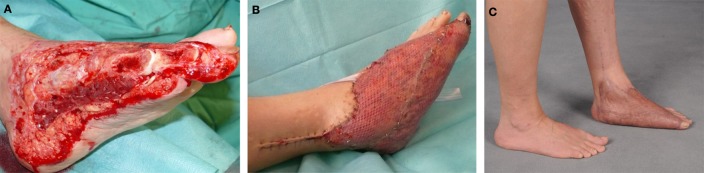
**A 46-year-old female with soft-tissue defect on her left foot after tumor resection (A)**. The reconstruction was carried out by free transplantation of a latissimus dorsi muscle with split skin **(A,B)**. Image of the foot after healing **(C)**.

If local flaps are not applicable for soft-tissue coverage, free tissue transfers can be utilized. Following the resection of a malignant tumor in the foot, the use of microvascularized tissue has been proven to be a successful surgical technique, offering an alternative to ablative surgery with functional restoration of the salvaged limb. Large defects of the foot can be treated by free microvascular myocutaneous or fasciocutaneous tissue transfer. If small defects, exposing bones or tendons, are not eligible for local flaps, small free microvascular flaps can be applied. These flaps cause a very low donor site morbidity. Myocutaneous and fasciocutaneous flaps have the advantage of replacing the missing tissue volume. Osseous surfaces can also be adequately covered and padded. If local flaps are not available, the implementation of a free tissue transfer should be taken into consideration. Such flaps provide adequate soft-tissue coverage for adjuvant radiation. A new group of propeller perforator flaps based on perforators from the anterior and posterior tibial artery and from the peroneal artery has been established in recent years, which also allows good defect management. In the Tokyo consensus, a propeller flap is defined as an island flap that reaches the recipient site through an axial rotation. The classification is based on the nourishing pedicle (subcutaneous pedicled propeller flap, perforator pedicled propeller flap, and supercharged propeller flap), the degrees of skin island rotation (from 90 to 180°), and, when possible, the artery of origin of the perforator (43, 44). Dong et al. recently reported a series of 20 patients with soft-tissue defects of the lower leg and foot that were covered by perforator pedicled propeller flaps (45). All flaps survived and the areas of soft-tissue defect ranged from 2 cm × 8cm to 10 cm × 20 cm. The donor sites could be closed primarily in 12 patients and skin grafted in 8 patients. Georgescu et al. reported another retrospectively analyzed series using perforator pedicled propeller flaps in 24 diabetic patients with acute and chronic wounds at the foot (46). A primary healing rate (96%) was obtained in 72% of all cases, whereas flap necrosis occurred in 24% and complete flap loss in 4%. Specific data after oncological resection and adjuvant therapy are still missing to date, but in experienced hands and in well selected cases, propeller perforator flaps can cover defects at the foot and ankle region reliably and provide an alternative to free flaps. In our experience, plastic reconstructive procedures after tumor resection in the foot and ankle area are required in about 50% of cases and are associated with a high success rate. Free transplants, such as fasciocutaneous flaps from the anterolateral thigh (ALT) and parascapular region, and also myocutaneous flaps, such as latissimus flap with or without a skin island, dominate. The ALT flap represents a versatile fasciocutaneous flap and is located at the anterolateral surface of the thigh (Figures 1 and 2). Its dominant pedicle proceeds from the septocutaneous or myocutaneous perforators of the descending branch of the lateral circumflex femoral artery. Primary closure of the donor site is usually possible when the flap width is 10 cm or less. Larger flaps need skin grafting of the donor site. Depending on the distribution of the subcutaneous fat in corpulent patients, the ALT flap might be less bulky than the parascapular flap in some cases and, thus, should be preferred in the soft-tissue reconstruction of the foot. The parascapular flap is a fasciocutaneous flap of the posterior trunk and is nourished by the descending branch of the circumflex scapular artery, which emerges from the triangular space between teres major, teres minor, and the long head of the triceps brachii muscle (Figures 3 and 4). The parascapular flap can be harvested up to 12 cm in width and 25 cm in length with primary closure. Similar to the parascapular flap, the latissimus dorsi muscle flap is also one of the most versatile flaps available to soft-tissue reconstruction (Figure 5). The expendable muscle with its reliable pedicle (thoracodorsal artery) can be harvested with or without a skin island from the posterior trunk and is excellently suited for widespread defects. The donor site morbidity of most free flaps is moderate and well tolerated by most of the patients, especially if the donor site can be closed primarily (47–49). Despite the adjuvant radiotherapy applied, the postoperative complications are tolerable and do not limit the use of microvascular tissue transfer.

As a reconstructive option after tumor resection, local flaps represent a reliable tool and can cover a wide range of smaller defects. Nevertheless, concerns over distant iatrogenic implantation of tumor cells at the donor site of local flaps exist when tumor resection and flap coverage were performed in the same surgical procedure. Reviewing the literature, there has, unfortunately, been no systematic analysis on this issue. However, iatrogenic tumor metastases at donor tissue sites after local flap reconstruction are a rare occurrence and should not preclude the use of local flap reconstruction (50). They have been described only in selected case reports (51). Furthermore, the effect of donor site radiation on the incidence of iatrogenic tumor metastases still remains unclear and should be examined (50). Beside the aforementioned concerns, local flaps can offer some slight but noteworthy advantages. In contrast to free flaps, local flaps do not require intensive postoperative flap inspections. Postoperative positioning protocols and anticoagulation regimens are less stringent. However, there has been a paradigm shift in the last few decades. Free tissue transfers can be performed with the same or even higher degree of safety than local flap transfer as a result of the improvements in microsurgical techniques nowadays. Safe dissection and positioning of a local flap at the hind foot can be technically more demanding, risky, and time-consuming when compared to a free flap transfer in a two-team approach. Due to the microsurgical and anesthesiological improvements, free flap transfers have become physically less demanding surgical procedures and have also become suitable for patients with ­vascular comorbidities.

Unfortunately, not all postoperative wound complications can be anticipated. For defects that extend to the tendons or joints, flap reconstruction is always required for adequate closure. A forced wound closure and “unexpected” wound complications can be slow to heal, devastating for the patient, and delay adjuvant radiotherapy. In this instance, early referral to a plastic surgeon is necessary in these cases to prevent devitalized superficial tissue from becoming infected and generating a deep infection that involves tendon, bone, or hardware. The use of free tissue transfer remains at the highest level on the reconstructive ladder. Although it is more complex, free tissue transfer may be necessary as a first choice. Our experience indicates that microvascularized tissue transfer is often the most suitable first option in accordance with established reconstructive principles. Furthermore, wound complications can occur more often after adjuvant radiation. Although defects, at first appearance, may be closed primarily or by using simple methods, such as split-thickness skin grafts, one must take the possibility of postoperative and postradiogenic wound complication into consideration. In those cases where there is an increased risk of exposure of tendons, bones or osteosynthetic material, a prophylactic use of an effective free flap for reconstruction should be considered. In the presence of avascular scar tissue, it should be removed before reconstruction. The use of prophylactic flaps in foot and ankle reconstruction is of great interest in patients with a high risk of wound healing disorders.

In addition to transplantation of tissue to cover the defects, the replacement of functional structures, such as tendons, vessels, and nerves, are at the forefront. Common peroneal nerve lesions or extensive loss of the anterior tibialis muscle might be an inevitable part of oncological resection and lead to a drop foot deformity. Primary transfer of the posterior tibialis tendon to the anterior tibialis tendon can restore the active extension of the foot (52, 53). There have been several technical modifications of this procedure to date and most of them improve the extremity function notably (54, 55). Indications for the oncological resection of the tibial or peroneal nerve are fortunately rare, but, if necessary, nerve grafting using the sural nerve can restore some protective touch sensibility at the plantar foot even after nerve defects of several centimeters (56, 57). However, there are still controversies about the timing of nerve grafting, especially when adjuvant radiation treatment is planned. There are missing specific data for peroneal and tibial nerve reconstructions to date, but a very recent study that analyzed the functional outcome of multiple sural nerve grafts for facial nerve defects after oncological resection could not detect any adverse effects of adjuvant radiation treatment on the outcome of the nerve grafts and endorsed an immediate nerve grafting after primary tumor resection (58).

## Conclusion

**Figure 6:**
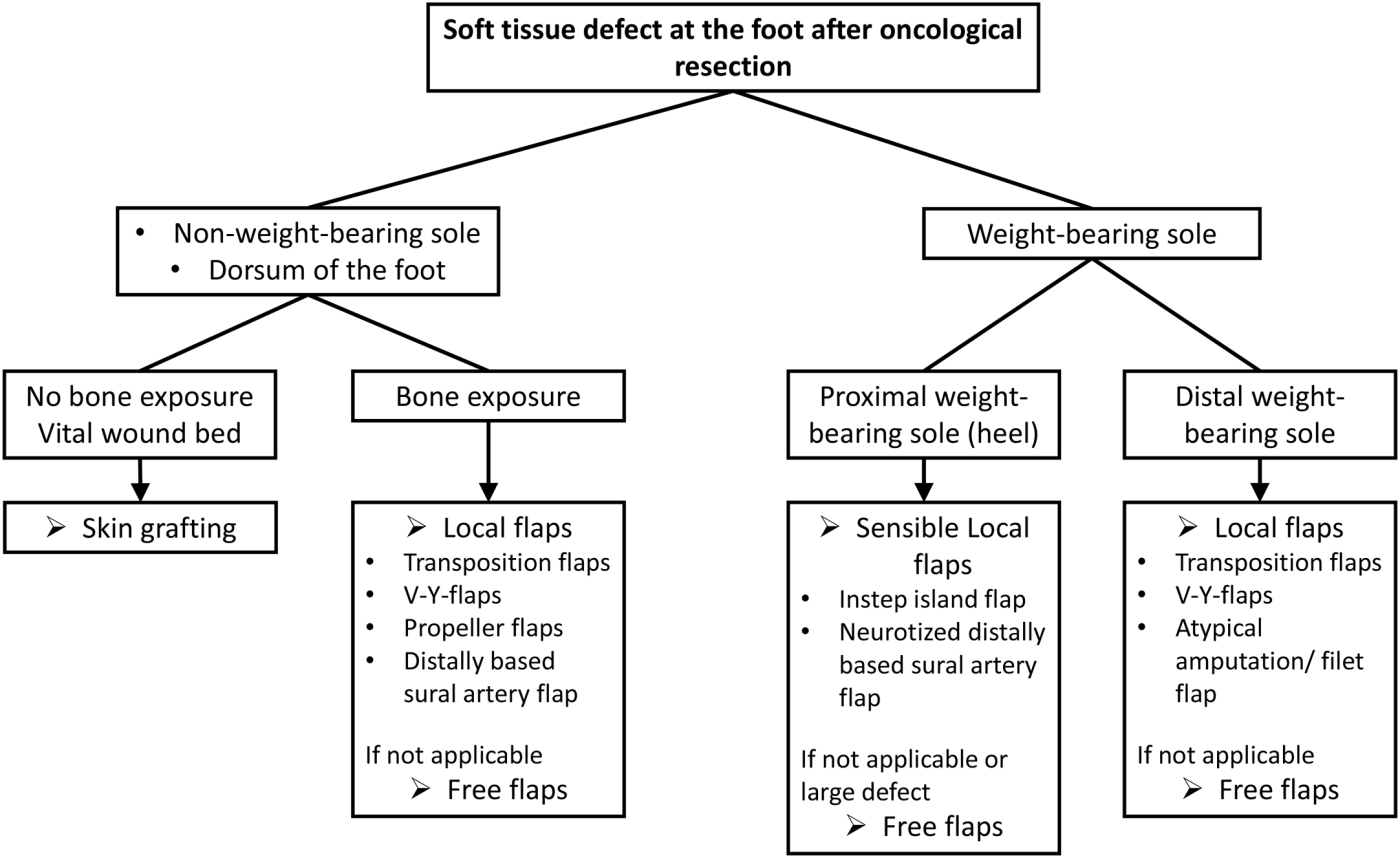
**Treatment algorithm for soft-tissue reconstructions at the foot after oncological resection**.

Surgical therapy provides the basis for local tumor control in soft-tissue sarcoma. The goal defined is the resection of the tumor with clear surgical margins, followed by adjuvant radiotherapy in highly malignant tumors. In many cases, more complex plastic surgical reconstructions, such as the replacement of nerves, blood vessels, and bone, or transfer of muscles or tendons, are required due to the close proximity to functionally relevant anatomical structures. Defects can be covered by means of plastic surgery techniques so that the foremost unresectable tumors become ­curable. In our institution, we use the treatment algorithm depicted in Figure 6 in order to cover most of the defects resulting after tumor resection.

At the present time, due to efforts in reconstructive plastic surgery, complex tumor resection with clear margins can be performed while still preserving the function that once may have been considered unsalvageable, particularly in the case of advanced tumors.

Patients are frequently presented with incompletely resected tumors. In such situations, the resection required is usually far more complex and extensive than the primary intervention. Due to this fact and the rarity and wide heterogeneity of soft-tissue sarcomas, they should be treated in specialized centers with plastic surgery as part of an interdisciplinary and multimodal treatment concept.

Our experience shows that reconstructive plastic surgery can play an integral role in the multimodal treatment concept including radiation and chemotherapy. The current plastic surgery techniques allow for the preservation and reconstruction of function after the resection of malignant tumors. The quality of life of the oncologic patient can be significantly improved by knowing and applying the opportunities of plastic reconstructive surgery.

## Author Contributions

AR and KH have written and prepared the manuscript. AD and ML reviewed and edited the manuscript. OG, PK, and BB helped to prepare the manuscript and the figures.

## Conflict of Interest Statement

The authors declare that the research was conducted in the absence of any commercial or financial relationships that could be construed as a potential conflict of interest.
